# The Lp_3561 and Lp_3562 Enzymes Support a Functional Divergence Process in the Lipase/Esterase Toolkit from *Lactobacillus plantarum*

**DOI:** 10.3389/fmicb.2016.01118

**Published:** 2016-07-19

**Authors:** María Esteban-Torres, Inés Reverón, Laura Santamaría, José M. Mancheño, Blanca de las Rivas, Rosario Muñoz

**Affiliations:** ^1^Laboratorio de Biotecnología Bacteriana, Instituto de Ciencia y Tecnología de Alimentos y Nutrición, ICTAN-CSICMadrid, Spain; ^2^Departamento de Cristalografía y Biología Estructural, Instituto de Química-Física “Rocasolano,” IQFR-CSICMadrid, Spain

**Keywords:** esterase, lipase, electrostatic potential surface, tandem duplication, paralog genes, lactic acid bacteria, genomic island

## Abstract

*Lactobacillus plantarum* species is a good source of esterases since both lipolytic and esterase activities have been described for strains of this species. No fundamental biochemical difference exists among esterases and lipases since both share a common catalytic mechanism. *L. plantarum* WCFS1 possesses a protein, Lp_3561, which is 44% identical to a previously described lipase, Lp_3562. In contrast to Lp_3562, Lp_3561 was unable to degrade esters possessing a chain length higher than C4 and the triglyceride tributyrin. As in other *L. plantarum* esterases, the electrostatic potential surface around the active site in Lp_3561 is predicted to be basic, whereas it is essentially neutral in the Lp_3562 lipase. The fact that the genes encoding both proteins were located contiguously in the *L. plantarum* WCFS1 genome, suggests that they originated by tandem duplication, and therefore are paralogs as new functions have arisen during evolution. The presence of the contiguous *lp_3561* and *lp_3562* genes was studied among *L. plantarum* strains. They are located in a 8,903 bp DNA fragment that encodes proteins involved in the catabolism of sialic acid and are predicted to increase bacterial adaptability under certain growth conditions.

## Introduction

Hydrolases constitute a class of enzymes that catalyze the hydrolysis of a wide variety of substrates. The diversity of substrate specificity has complicated hydrolase classification. However, they are typically classified to their known specificity. Among hydrolases, esterases (EC 3.1.1) hydrolyze ester bonds and are subdivided as carboxylesterases (EC 3.1.1.1, true esterases), when they catalyze the hydrolysis of small carboxylic acid ester-containing molecules at least partially soluble in water, or lipases (EC 3.1.1.3), when maximal hydrolytic activity is displayed against water-insoluble long chain triglycerides (Arpigny and Jaeger, 1999). Although an operational distinction is made between carboxylesterases and lipases, no fundamental chemical difference exists (Bornscheuer, 2002). From a structural point of view, both carboxylesterases and lipases are members of the α/β hydrolase superfamily and share a common catalytic machinery for ester hydrolysis and formation, which is based on a catalytic triad (Bornscheuer, 2002). Classifications based on sequence similarities do not separate the two classes of enzymes. The definite approach to assign a specific molecular function to a predicted open reading frame is to biochemically characterize the corresponding protein, mainly the substrate specificity.

Lactic acid bacteria are extensively used for the fermentation of food products. Among lactic acid bacteria, *Lactobacillus plantarum* is an industrially important species, which can be found in numerous fermented foods (Kleerebezem et al., 2003). This species possesses a versatile metabolism and is a good source of esterases to produce relevant metabolites that affect to the organoleptic characteristics of fermented foods. Both lipase and esterase activities have been previously described in *L. plantarum* strains (Oterholm et al., 1967, 1968, 1972; Silva Lopes et al., 1999). Although numerous genome sequences from *L. plantarum* are currently available, there is still limited information on the function of genes predicted to encode esterases. In this regard, a wide study to dissect the complex array of esterase activities in *L. plantarum* WCFS1 was previously undertaken. In that study 9 esterases from *L. plantarum* WCFS1 were recombinantly produced and biochemically characterized. These esterase proteins exhibited diverse specific activities, such as feruloyl esterase (Lp_0796; Esteban-Torres et al., 2013), arylesterase (Lp_1002 and Lp_2631; Esteban-Torres et al., 2014a,c), carboxylesterase (Lp_0973 and Lp_2923; Benavente et al., 2013; Álvarez et al., 2011, 2014), acetylesterase (Lp_3505; Esteban-Torres et al., 2014d), tributyrin esterase (Lp_1760; Esteban-Torres et al., 2014b), and lipase (Lp_3562; Esteban-Torres et al., 2015b). In the present work, *lp_3561*, a new esterase in *L. plantarum* WCFS1, was identified and characterized.

## Materials and Methods

### Bacterial Strains and Growth Conditions

Twenty eight strains of *L. plantarum* were used in this study. *L. plantarum* WCFS1 (a colony isolate of *L. plantarum* NCIMB 8826 that was isolated from human saliva), NC8, and LPT 57/1 strains were kindly provided by M. Kleerebezem (NIZO Food Research, The Netherlands), L. Axelsson (Norwegian Institute of Food, Fisheries and Aquaculture Research, Norway), and J. L. Ruíz-Barba (Instituto de la Grasa, CSIC; Spain), respectively. Eight strains were provided by the Spanish Type Culture Collection (CECT): *L. plantarum* CECT 220 (ATCC 8014), CECT 221 (ATCC 14431), CECT 223, CECT 224, CECT 749 (ATCC 10241), CECT 4645 (NCFB 1193), and the type strain *L. plantarum* subsp. *plantarum* CECT 748^T^ (ATCC 14917, DSMZ 20174). Seven strains were purchased from the German Collection of Microorganisms and Cell Cultures (DSMZ): *L. plantarum* DSM 1055, DSM 2648, DSM 10492, DSM 12028, DSM 13273, DSM 20246, and the type strain of *L. plantarum* subsp. *argentoratensis* DSM 16365^T^. Eleven strains were isolated from must grape or wine of different wine-producing areas of Spain over the period from 1998 to 2001 (*L. plantarum* RM28, RM31, RM34, RM35, RM38, RM39, RM40, RM41, RM71, RM72, and RM73; Moreno-Arribas et al., 2003).

*Lactobacillus plantarum* strains were grown in MRS medium (Pronadisa, Spain) adjusted to pH 6.5 and incubated at 30°C. *Escherichia coli* DH10B (Invitrogen, Warrington, UK) was used as host strain for all DNA manipulations. *E. coli* BL21 (DE3) was used for heterologous expression in the pURI3-Cter vector (Curiel et al., 2011). *E. coli* strains were cultured in Luria-Bertani (LB) medium at 37°C with shaking at 200 rpm. When required, ampicillin and chloramphenicol were added at a concentration of 100 or 20 μg/ml, respectively.

### PCR Detection of *lp_3561* and *lp_3562* Genes

Genomic DNA from *L. plantarum* strains was extracted as previously described (Vaquero et al., 2004). Genes encoding *L. plantarum* Lp_3561 and Lp_3562 esterases (*lp_3561* and *lp_3562*) were amplified by PCR using chromosomal DNA. The *lp_3561* gene (0.8 kb) was amplified by using primers 957 (5′-TAACTTTAAGAAGGAGATATACATATGAGATATGAGCAATTGAGATTAA) and 958 (5′-GCTATTAATGATGATGATGATGATGTTTAAAGTCCACTTGCGTCAAATC). Oligonucleotides 575 and 576 were used to amplify *lp_3562* gene (0.8 kb; Esteban-Torres et al., 2015b). The reactions were performed in a Mastercycler personal thermal cycler (Eppendorf) using 30 cycles of denaturation at 94°C for 30 s, annealing at 50°C for 1 min, and extension at 72°C for 1 min.

### Production and Purification of Lp_3561

The gene encoding a putative esterase/lipase (*lp_3561*, accession YP_004891038.1) in *L. plantarum* WCFS1 was amplified by PCR using the primers 957 and 958. Prime Star HS DNA polymerase (TaKaRa) was used for PCR amplification. The 837-bp purified PCR product was inserted into the pURI3-Cter vector using a restriction enzyme- and ligation-free cloning strategy (Curiel et al., 2011). This vector produces recombinant proteins having a six-histidine affinity tag at their *C*-terminal ends. *E. coli* DH10B cells were transformed and, for expression, the recombinant plasmid obtained (pURI3-Cter-3561) was transformed into *E. coli* BL21 (DE3) with pGro7 (TaKaRa), a vector overexpressing GroES/GroEL chaperones. The recombinant Lp_3561 enzyme was produced as previously described for esterase Lp_2631 (Esteban-Torres et al., 2014c), and purified by immobilized metal affinity chromatography (IMAC) using a Talon Superflow resin (Esteban-Torres et al., 2013).

### Esterase Activity and Substrate Specificity Assays

Esterase activity was determined by, a spectrophotometric method previously described using *p*-nitrophenyl acetate (Sigma–Aldrich, Steinheim, Germany) as substrate (Esteban-Torres et al., 2013). The substrate specificity of Lp_3561 was determined using different *p*-nitrophenyl esters of various chain lengths (Sigma–Aldrich): *p*-nitrophenyl acetate (C2), *p*-nitrophenyl butyrate (C4), *p*-nitrophenyl caprylate (C8), *p*-nitrophenyl laurate (C12), *p*-nitrophenyl myristate (C14), and *p-*nitrophenyl palmitate (C16) as substrates as described previously (Brod et al., 2010; Esteban-Torres et al., 2013). The substrate profile of Lp_3561 was also determined by using an ester library described previously (Esteban-Torres et al., 2013).

Esterase activity was assayed in the pH range from 3.0 to 9.0, and at temperatures of 5, 20, 30, 37, 40, 45, 55, and 65°C as described previously (Esteban-Torres et al., 2013). Enzyme thermostability was measured by incubation of the enzyme in 50 mM sodium phosphate buffer (pH 7.0) at 20, 30, 37, 45, 55, and 65°C for 5 min, 15 min, 30 min, and 1, 2, 4, 6, and 20 h. After incubation, the residual activity of Lp_3561 was measured as described above. To test the effects of metals, ions and additives on the activity of the esterase, Lp_3561 was incubated in their presence at a final concentration of 1 mM for 5 min at room temperature. Then, the substrate (*p*-nitrophenyl acetate) was added, and the reaction mixture was incubated at 37°C. The experiments were performed in triplicate.

### Modeling of the Structures of Lp_3561 and Lp_3562

The 3D structures of Lp_3561 and Lp_3562 were modeled with the Swiss-Model server^1^ (Biasini et al., 2014). The obtained atomic coordinates were then subjected to geometry minimization with the tool geometry minimization from Phenix (Adams et al., 2010). Validation of the structures was done with Molprobity (Chen et al., 2010).

## Results

### Tandem Duplication as a Possible Origin of *lp_3561*

**FIGURE 1:**
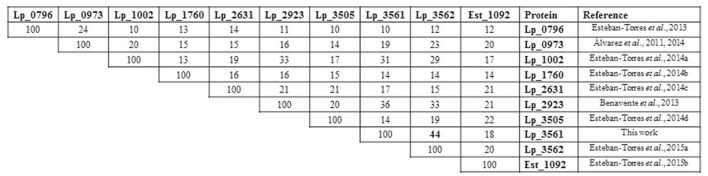
**Identity degree among *Lactobacillus plantarum* WCFS1 characterized esterases.** Protein alignments were performed by using ClustalW2 at the EBI site (http://www.ebi.ac.uk/Tools/msa/clustalw2/). Protein sequences are Lp_0796 (accession YP_004888771), Lp_0973 (YP_004888920), Lp_1002 (YP_004888940), Lp_1760 (YP_004889565), Lp_2631 (YP_004890283), Lp_2923 (YP_004890513), Lp_3505 (YP_004890987), Lp_3561 (YP_004891038), Lp_3562 (YP_004891039), and Est_1092 (ACT61979).

**FIGURE 2:**
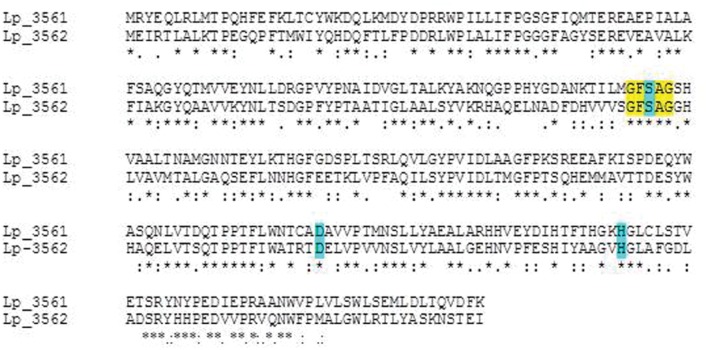
**Amino acid sequence alignment of Lp_3561 (YP_004891038) and Lp_3562 (YP_004891039) esterases from *L. plantarum* WCFS1 strain.** Alignments were done by using the ClustalW2 program at the EBI site (http://www.ebi.ac.uk/Tools/msa/clustalw2/). The serine hydrolase conserved motif is highlighted in yellow. Residues forming the catalytic triad (Ser-116, Asp-201, and His-233) are blue-highlighted in the figure.

A protein amino acid sequence alignment of the nine esterases characterized in *L. plantarum* showed that only a 10–33% identity is found among them, being not related with their esterase activity on specific substrates (**Figure 1**). However, among the *L. plantarum* WCFS1 proteins annotated as “esterase/lipase” in its genome, the highest sequence identity is shown between Lp_3561 and Lp_3562 (44%). Both proteins share other additional features: they are 278 amino acid proteins, have a similar theoretical isoelectric point of 5.4 and 5.1, and molecular sizes of 31.5 and 30.9 kDa, for Lp_3561 and Lp_3562, respectively. According to the ESTHER database Lp_3561 (lacpl-LP.3561) and Lp_3562 (lacpl-LP.3562), belong to the hormone-sensitive lipase family (block H; family IV according to Arpigny and Jaeger, 1999). Surprisingly, the genes encoding these proteins exhibited higher identity than the corresponding encoded proteins among them (58% vs. 44%; **Figure 2**). Moreover, both genes (*lp_3561* and *lp_3562*) are only separated by 13 bp, which points to a process of gene tandem duplication.

### Production and Biochemical Characterization of Lp_3561 Esterase

**FIGURE 3:**
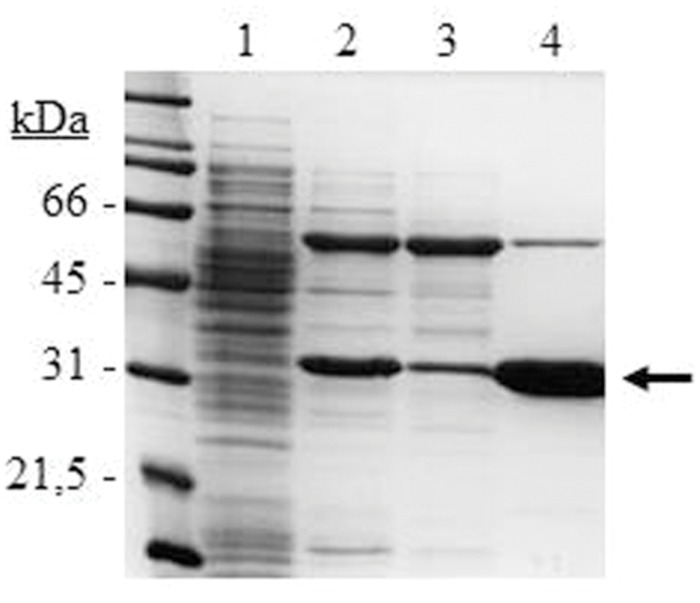
**SDS-PAGE analysis of the expression and purification of *L. plantarum* WCFS1 Lp_3561 protein.** Analysis of soluble cell extracts of IPTG-induced *Escherichia coli* BL21(DE3) (pURI3-Cter) (1) or *E. coli* BL21(DE3) (pURI3-Cter-3561) (pGro7) (2), flowthrough (3), or fraction eluted from the His affinity resin (4). The arrow indicated the overproduced and purified protein. The gel was stained with Coomassie blue. Molecular mass markers are located at the left (SDS-PAGE Standards, Bio-Rad).

The *lp_3561* gene from *L. plantarum* WCFS1 was cloned into the pURI3-Cter vector (Curiel et al., 2011) and transformed into *E. coli* BL21 (DE3) cells. SDS-PAGE analysis of cell extracts showed a major protein band of approximately 30 kDa, present as inclusion bodies in the insoluble fraction (data not shown). To obtain Lp_3561 in a soluble form co-overexpression with molecular chaperones was considered by using the plasmid pGro7 as previously published (Esteban-Torres et al., 2015a). When pURI3-Cter-3561 and pGro7 were used simultaneously, Lp_3561 appeared in the intracellular soluble fraction of the cells (**Figure 3**). Lp_3561 was purified by IMAC.

**FIGURE 4:**
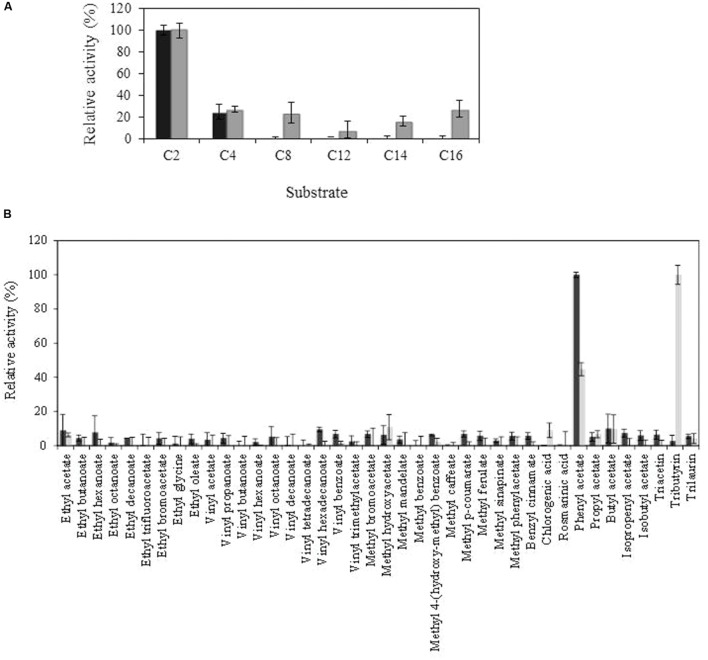
**Comparison of Lp_3561 and Lp_3562 substrate profile, against chromogenic substrates (*p*-nitrophenyl esters) with different acyl chain lengths (C2, acetate; C4, butyrate C8, caprylate; C12, laurate; C14, myristate; C16, palmitate) **(A)** or toward a general ester library **(B)**.** Lp_3561 is represented by black bars, and Lp_3562 by gray bars. The error bars represent the standard deviation estimated from the three independent assays. The observed maximum activity was defined as 100%.

Purified Lp_3561 protein was biochemically characterized. Esterase activity was determined using *p*-nitrophenyl esters possessing acyl chains with different lengths from C2 to C16. From the substrates assayed, Lp_3561 showed preference for *p*NP-acetate, being unable to degrade esters with chain lengths higher than C4 (**Figure 4A**). This result was also confirmed when the esterase activity was assayed on a library composed of 40 different esters (**Figure 4B**). From the esters assayed, only phenyl acetate, a short acyl chain ester, was efficiently hydrolyzed by Lp_3561.

**FIGURE 5:**
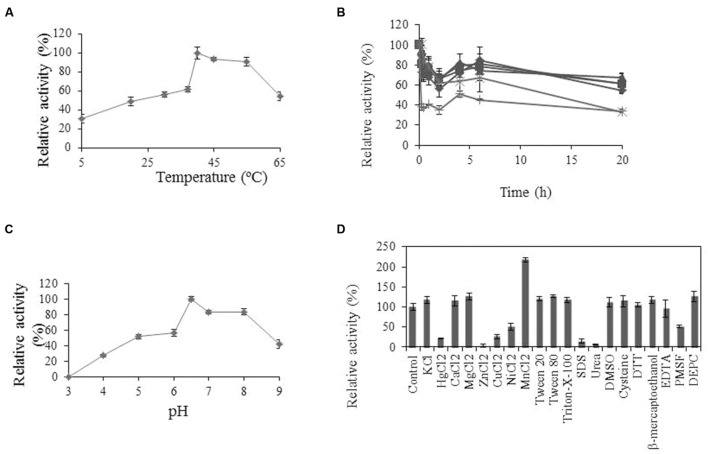
**Biochemical properties of Lp_3561 esterase.** **(A)** Temperature-activity profile; **(B)** thermal stability profile after pre-incubation (relative to unincubated enzyme) at 20°C (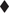), 30°C (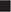), 37°C (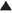), 45°C (x), 55°C (^∗^), and 65°C (●). **(C)** pH-activity profile. At the indicated times, aliquots were withdrawn and analyzed. The experiments were done in triplicate. The mean values and standard errors are shown. The observed maximum activity was defined as 100%. **(D)** Relative activities of Lp_3561 after incubation with 1 mM concentrations of different additives. The activity of the enzyme incubated in the absence of additives was defined as 100%.

Some physicochemical properties of Lp_3561 were also analyzed. Esterase Lp_3561 showed maximal activity at pH 6.5 and 40°C (**Figure 5**). Lp_3561 retained 40% of its maximal activity at 55 and 65°C after prolonged incubation time. Activity was greatly increased by the addition of MnCl_2_ and inhibited by ZnCl_2_, urea and SDS.

### Structural Models of Lp_3561 and Lp_3562 Enzymes

**FIGURE 6:**
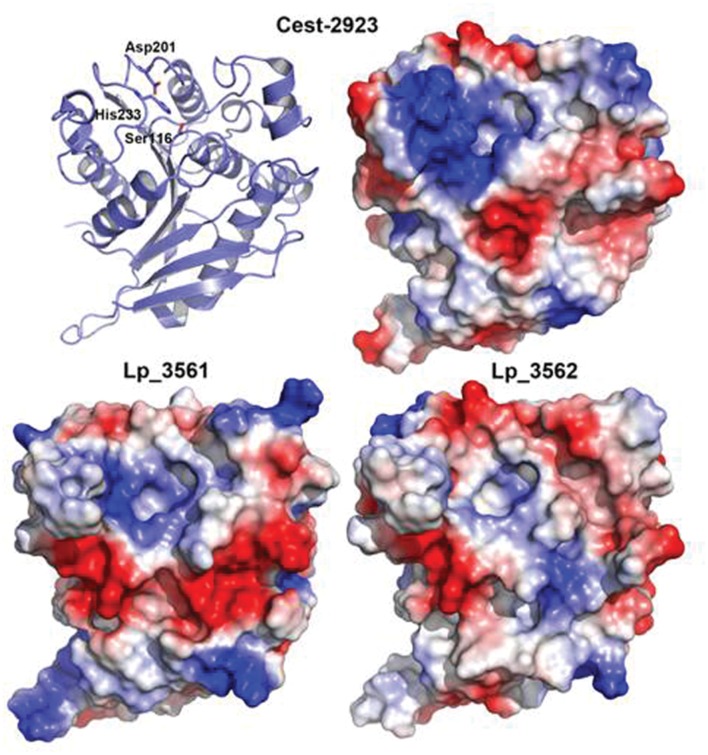
**Comparison of the electrostatic potential surfaces of the 3D structural models of Lp_3561 (*lower left*) and Lp_3562 (*lower right*) prepared with the Swiss-Model server with that from Cest-2923 from *L. plantarum* (PDB entry: 4bzw; *upper right*).** The polypeptide backbone of Cest-2923 is shown as *ribbon* model (*upper left*) to indicate the orientation of the molecules. Amino acid residues forming the catalytic triad (Ser-116, Asp-201, and His-233) are shown as *sticks* to identify the location of the active site. The figure has been prepared with *PyMOL* (DeLano, 2008). Red color indicates negative potential (excess of negative charges) and blue color indicates positive potential (excess of positive charges).

With the aim of characterizing the 3D structure of Lp_3561 we carried out crystallization trials using the same experimental setup for Cest-2923 (Benavente et al., 2013). Unfortunately, after exhaustive screening for crystallization conditions no positive results were obtained. As an indirect approach, we have modeled the 3D structures of Lp_3561 and Lp_3562 by using the Swiss-Model server, which interestingly, found as template for both proteins the atomic coordinates of the esterase Cest-2923 (Lp_2923) from *L. plantarum* WCFS1 (PDB entry: 4bzw) that we have determined recently (Benavente et al., 2013). Cest_2923 shares a 36% sequence identity to Lp_3561 and 33% to Lp_3562. The 3D structures of both enzymes show the characteristic α/β hydrolase fold. The structural models of Lp_3561 and Lp_3562 revealed the presence of the catalytic triad (Ser-116, Asp-201, and His-233). The serine residue appears in the conserved pentapeptide Gly-Phe-Ser-Ala-Gly. Nonetheless, more interesting results are provided by the analysis of the electrostatic potential surfaces, in particular, the electrostatic features in regions around the active sites (**Figure 6**), which are known to affect to the esterase/lipase character of the enzymes (Fojan et al., 2000). As indicated below in detail, Lp_3561 and Cest-2923 share characteristics such as a basic region close to the active site together with an acidic crevice besides the active site, which are not clearly defined in Lp_3562.

### Lp_3561 Esterase and Lp_3562 Lipase are Linked to Genes Involved in Sialic Acid Metabolism in *L. plantarum*

**FIGURE 7:**
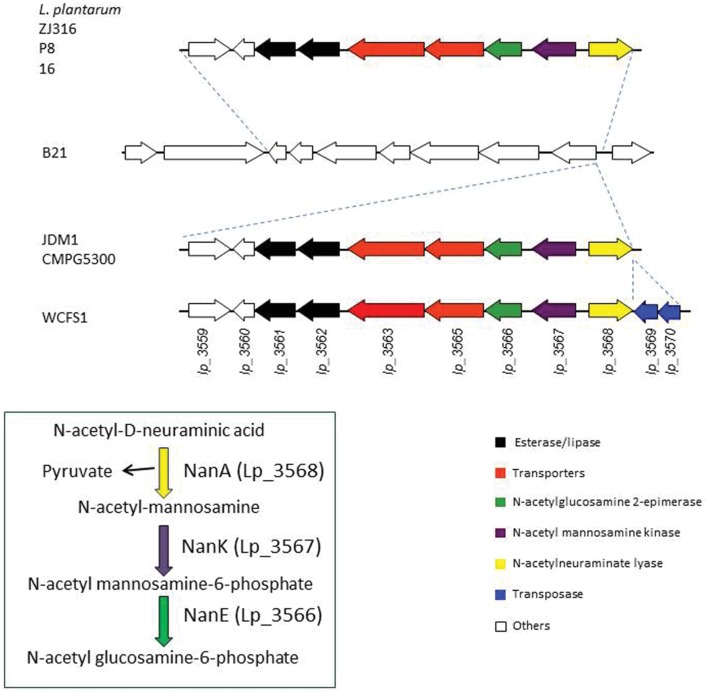
**Schematic overview of the genetic organization of the chromosomal region containing paralog Lp_3561 and Lp_3562 esterases in sequenced *L. plantarum* strains: P8 (accession number CP005942.2, positions 2958537-2972900), Zj316 (accession number NC_020229.1, positions 177.896-192.259), 16 (accession number NC_021514.1, positions 2970898-2985261), B21 (accession number NZ_CP010528.1, positions 3150752-3164323), JDM1 (accession number NC_012984.1, positions 3062356-3084735), CMPG5300 (accession number CM002918.1, positions 3097771-3120078), and WCFS1 (accession number NC_004567.2, positions 3168151-3120078).** Genes are represented by arrows. Genes coding for paralog esterases are represented by black arrows.

The genomes of several *L. plantarum* strains are currently available. Analyses of these genomes revealed that, when present, the copies of *lp_3561* and *lp_3562* genes are always adjacent; however, both genes are absent in numerous strains, e.g., on the *L. plantarum* type strain (ATCC 14917^T^) both genes are absent. In order to determine the extent of the presence of both paralog genes among strains belonging to the *L. plantarum* group, their presence was studied in 28 *L. plantarum* strains isolated from different sources. To determine the presence of both genes, chromosomal DNA was extracted and PCR amplified using oligonucleotides designed on the basis of the *L. plantarum* WCFS1 sequence. Apart from *L. plantarum* WCFS1, ten additional strains possessed *lp_3561* and *lp_3562* genes [*L. plantarum* 57/1, CECT 220, CECT 221, CECT 749 (ATCC 10241), DSM 13273, RM28, RM31, RM35, RM38, and DSM 26365; data not shown]. Interestingly, all the strains which possess one of these genes possessed also the other gene. This observation was also noticed on the *L. plantarum* strains whose complete genome is available. Moreover, in these strains both genes are contiguous. For example, on *L. plantarum* ZJ316 strain, *lp_3561* is located on the *zj316_0167* locus and *lp_3562* is located contiguously on the *zj316_0168* locus. A more detailed analysis of the *L. plantarum* strains whose genomes are available identified a 8,903 bp region only present in the strains possessing both esterase genes (**Figure 7**). The publically available genomes of seven *L. plantarum* strains revealed that this region is only absent on *L. plantarum* B21 strain. This allowed for identification of insertion point at the 8,903 region on the intergenic region within the SH83_RS14770 locus (encoding a GntR family transcriptional regulator) and SH83-RS14775 locus (encoding a *N*-acetylmannosamine-6-phosphate 2-epimerase). Strains JDM1 and CMPG5300 possessed identical organization, while WCFS1 strain possessed an additional 854 bp region encoding two putative transposases (Lp_3569 and Lp_3570). In several *L. plantarum* strains (P8, Zj316, and 16), the insertion of this 8,903 bp region has been accompanied with the deletion of seven genes (from SH83_RS14740 to SH83_RS14770 locus in *L. plantarum* B21; **Figure 7**).

In addition to the two esterase genes (*lp_3561* and *lp_3562* in WCFS1 strain), the 8,903 pb region encoded proteins involved in the catabolism of *N*-acetyl-D-neuraminic acid, a sialic acid (Lp_3566 to Lp_3568).

## Discussion

In fermented foods, microorganisms are in contact with food substrates and their metabolic activities influence food aroma. Numerous ORFs encoding putative esterases/lipases have been found in the genome of the food bacterium *L. plantarum* WCFS1 strain. The catalytic machinery of these proteins is based on a catalytic triad formed by the residues Ser-Asp-His, which follow the order Ser-Asp-His. The nucleophile is located in a consensus sequence motif (Gly-x-Ser-x-Gly). Apart from these two motifs, these proteins exhibited low sequence similarity. The nine esterases characterized in *L. plantarum* WCFS1 showed that only a 10–33% identity is found among them, being the highest sequence identity found between Lp_3561 and Lp_3562 (44%). These two proteins also shared additional features that may suggest that both genes could be originated by tandem duplication (Reams and Neidle, 2004). Gene duplication provides the opportunity for increased gene content and specialization of the divergent enzymes (Jensen, 1976).

A detailed comparison of the amino acid sequences of Lp_3561 and Lp_3562 with those from members of different lipase families (Arpigny and Jaeger, 1999) revealed insights into potential functional divergence between them. Both proteins posses the characteristic pentapeptide signature around the putative nucleophile Ser116 (GxSxG), the sequence following this pentapeptide in Lp_3562 closely matches the motif GxLA(A/L) (GHLVA), which is also typical of the family IV type lipases, in contrast to the sequence in Lp_3561 (SHVAA), which clearly diverges from it. Additionally, the residues Pro192 and Ile195 in Lp_3562, situated in close proximity to the catalytic Asp201, are strictly conserved in bacterial members from family IV lipases; however, in Lp_3561 Ile195 is substituted for Leu195. Moreover, the sequence following this catalytic Asp201 residue in Lp_3561 (DAVVP) is conserved in the family VI members (DxVVP) and not in lipases from family IV (the Lp_3562 sequence is DELVP). As a whole, these features reveal amino acid changes between Lp_3561 and Lp_3562, in positions that contribute to the definition of different esterase/lipase families (Arpigny and Jaeger, 1999) that support a functional divergence between these two proteins.

In order to know whether both enzymes (Lp_3561 and Lp_3562) exhibited also similar biochemical properties, the biochemical activity of Lp_3561 was determined and compared to Lp_3562 which exhibited lipase activity (Esteban-Torres et al., 2015b). The *lp_3561* gene from *L. plantarum* WCFS1 and purified the recombinant Lp_3561 protein produced and biochemically characterized. Esterase activity was determined using *p*-nitrophenyl esters possessing acyl chains with different lengths. From the substrates assayed, Lp_3561 showed preference for *p*NP-acetate, being unable to degrade esters with chain lengths higher than *p*NP-butyrate (C4). This contrasts with the results obtained for Lp_3562, which was active against all the substrates assayed, from C2 to C16 (Esteban-Torres et al., 2015b). By using a 40 esters library, only phenyl acetate, a short acyl chain ester, was efficiently hydrolyzed by Lp_3561; however, despite phenyl acetate was also hydrolyzed by Lp_3562, the triglyceride tributyrin was the substrate most efficiently hydrolyzed by Lp_3562 (Esteban-Torres et al., 2015b). Tributyrin is a true fat and the simplest triglyceride occurring in natural fats and oils. It is a common constituent of lipase testing media as it is easily dispersed in water (Samad et al., 1989). Lipases prefer water-insoluble substrates, typically triglycerides composed of long-chain fatty acids, whereas esterases preferentially hydrolyze “simple esters” (e.g., ethyl acetate). The obtained results clearly indicated that Lp_3561 did not possess the lipase activity exhibited by Lp_3562. Most probably, the *lp_3561* and *lp_3562* genes originated by tandem duplication, then, once the duplicated genes have diverged sufficiently, new functions can arise.

Comparison of amino acid sequences and 3D-structures of lipases and esterases suggested that they can be distinguished by a pH-dependent electrostatic “signature” (Fojan et al., 2000); the active site of lipases displays a negative potential in the pH-range associated with their maximum activity, whereas esterases show a similar pattern, but at pH values around 6, which correlates with their usually lower pH-activity optimum (Fojan et al., 2000). Esterase Lp_3561 showed an optimum pH of pH 6.5, which is lower than its lipase paralog Lp_3562 (pH 7.0). Lp_3561 also showed hydrolytic activity in a broader pH range than Lp_3562 (Esteban-Torres et al., 2015b).

As indicated above, interesting information can be inferred from the analysis of the main features of the electrostatic potential surfaces of these proteins around the putative active sites. Notably, these features are more similar between Lp_3561 and Cest-2923 than between Lp_3562 and Cest-2923, which is consistent with the fact that the former two proteins share essentially the same substrate specificity profiles, namely, both are esterases. In particular, in both proteins it is observed a basic region close to the active site (essentially neutral in Lp_3562) together with a neighboring acidic crevice, which is basic in Lp_3562. Whereas the residues of Lp_3561 contributing to the basic character of the above region around the active site (distances <13 Å) are Arg52, Lys232, Arg244, and Glu249, those from Lp_3562 are Arg52, Arg244, and Glu249 (Arg51, Lys244, Lys247, and Lys249 would be the equivalent residues in Cest-2923). Conversely, the acidic residues present in the crevice of Lp_3561 are Glu15, Glu51, Glu55, and Glu72, whereas those from Lp_3562 are Glu55, Lys72, and Glu78 (in Cest-2923 the residues are Glu53 and Asp78). Since these electrostatic features may relevant in conferring the esterase character to Lp_3561 in contrast to the lipase character of Lp_3562, the role of these differential residues are currently being explored, in particular Lys72 in Lp_3562 which breaks the basic character of the crevice. In this regard, we would like to remark that despite the above molecular characteristics are derived from theoretical, structural models we believe that there are two aspects that further support our conclusions. First, the α/β hydrolase fold is a highly conserved protein architecture of the lipase/esterase superfamily (Heikinheimo et al., 1999). Members from this group of enzymes sharing low sequence identities possess essentially the same structure, the differences being mainly located in loops far from the ligand-binding site (see Benavente et al., 2013). Second, the obtained models after geometry minimization show good geometry according to the validation process with MolProbity.

Tandem gene amplification occurs frequently and allows adaptation to a wide variety of conditions (Reams and Neidle, 2004). The study of the presence of *lp_3561* and *lp_3562* genes in *L. plantarum* strains revealed that these genes are frequently absent on strains from this bacterial species. Generally, these genes are inserted as a 8903 bp region contiguous to the genes *nanAKE* (*lp_3568*, *lp_3576*, and *lp_3566*) that encode proteins involved in the catabolism of *N*-acetyl-D-neuraminic acid, the most abundant and widely studied sialic acid (Tanner, 2005; Tao et al., 2010). Sialic acid catabolism has been found in some bacteria which use *N*-acetyl-D-neuraminic acid as a carbon and nitrogen source by scavenging it from the mucus-rich environment (Almagro-Moreno and Boyd, 2009). Noteworthy, apart from a few aquatic bacteria, the *nanA* gene is present only in commensal or pathogenic bacteria related with humans (Almagro-Moreno and Boyd, 2009). Interestingly, the presence of both paralog esterases in *L. plantarum* is genetically associated with the catabolism of sialic acid; in fact, a detailed examination of the *L. plantarum* B21 genome, the sequenced strain lacking paralog esterase genes, revealed that this strain is also devoid of the genes involved in the catabolism of sialic acid.

In this context, the biological relevance of the genes coding for both esterases (*lp_3561* and *lp_3562*) is still unknown. A plausible hypothesis explaining their presence next to the cluster of the *nanAKE* genes is that they would play the role of NagA, which is not present in the *L. plantarum* strains that acquired the *nanAKE* genes by horizontal transfer. As expected from this hypothesis, we observe that Lp_3561 and Lp_3562 hydrolyze ester bonds with acetyl groups in the acidic part similarly to NagA.

Paralog genes are often present as a tandem in prokaryotic genomes. Some of paralog genes are on genomic islands, which are likely to have been horizontally acquired, to be highly polymorphic among strains, and to confer strain-specific adaptative properties (Tsuru and Kobayashi, 2008). A typical genomic island carries genes encoding traits that may increase bacterial adaptability under certain growth conditions. Therefore, the 8,903 bp region containing paralog esterases Lp_3561 and Lp_3562 may be a genomic island acquired by horizontal gene transfer in some *L. plantarum* strains.

## Author Contributions

Substantial contributions to the conception or design of the work (RM, ME, BR); acquisition, analysis or interpretation of data for the work: gene cloning, protein production and substrate range studies (ME), protein biochemical characterization (LS), PCR experiments (IR), and protein modeling (JM). Drafting the work or revising it critically for important intellectual content (ME, IR, LS, JM, BR, RM). Final approval of the version to be published (ME, IR, LS, JM, BR, RM). Agreement to be accountable for all aspects of the work in ensuring that questions related to the accuracy or integrity of any part of the work are appropriately investigated and resolved (ME, IR, LS, JM, BR, RM).

## Conflict of Interest Statement

The authors declare that the research was conducted in the absence of any commercial or financial relationships that could be construed as a potential conflict of interest.

## Footnotes

1 www.swissmodel.expasy.org
